# Distinct SARS-CoV-2 RNA fragments activate Toll-like receptors 7 and 8 and induce cytokine release from human macrophages and microglia

**DOI:** 10.3389/fimmu.2022.1066456

**Published:** 2023-01-13

**Authors:** Thomas Wallach, Martin Raden, Lukas Hinkelmann, Mariam Brehm, Dominik Rabsch, Hannah Weidling, Christina Krüger, Helmut Kettenmann, Rolf Backofen, Seija Lehnardt

**Affiliations:** ^1^ Institute of Cell Biology and Neurobiology, Charité – Universitätsmedizin Berlin, Corporate Member of Freie Universität Berlin, Humboldt-Universität zu Berlin, and Berlin Institute of Health, Berlin, Germany; ^2^ Bioinformatics, Department of Computer Science, Albert-Ludwigs-University Freiburg, Freiburg, Germany; ^3^ Cellular Neuroscience, Max Delbrueck Center for Molecular Medicine in the Helmholtz Association, Berlin, Germany; ^4^ Signalling Research Centres BIOSS and CIBSS, University of Freiburg, Freiburg, Germany; ^5^ Department of Neurology, Charité – Universitätsmedizin Berlin, Corporate Member of Freie Universität Berlin, Humboldt-Universität zu Berlin, and Berlin Institute of Health, Berlin, Germany

**Keywords:** SARS-CoV-2, RNA, toll-like receptors, iPSC-derived human microglia, macrophages, inflammatory response

## Abstract

**Introduction:**

The pandemic coronavirus disease 19 (COVID-19) is caused by severe acute respiratory syndrome coronavirus 2 (SARS-CoV-2) and is marked by thromboembolic events and an inflammatory response throughout the body, including the brain

**Methods:**

Employing the machine learning approach BrainDead we systematically screened for SARS-CoV-2 genome-derived single-stranded (ss) RNA fragments with high potential to activate the viral RNA-sensing innate immune receptors Toll-like receptor (TLR)7 and/or TLR8. Analyzing HEK TLR7/8 reporter cells we tested such RNA fragments with respect to their potential to induce activation of human TLR7 and TLR8 and to activate human macrophages, as well as iPSC-derived human microglia, the resident immune cells in the brain.

**Results:**

We experimentally validated several sequence-specific RNA fragment candidates out of the SARS-CoV-2 RNA fragments predicted in silico as activators of human TLR7 and TLR8. Moreover, these SARS-CoV-2 ssRNAs induced cytokine release from human macrophages and iPSC-derived human microglia in a sequence- and species-specific fashion.

**Discussion:**

Our findings determine TLR7 and TLR8 as key sensors of SARS-CoV-2-derived ssRNAs and may deepen our understanding of the mechanisms how this virus triggers, but also modulates an inflammatory response through innate immune signaling.

## Introduction

The severe acute respiratory syndrome coronavirus-2 (SARS-CoV-2)-induced pneumonia outbreak in 2019 is the cause of the current coronavirus disease 2019 (COVID-19) pandemic. While most SARS-CoV-2 infections, which primarily affect the respiratory tract, remain mild, or even are asymptomatic, some patients with SARS-CoV-2 infection suffer severe disease and develop systemic inflammation, thereby suffering disorders of multiple organs, including the brain (1–3). Mild neurological complications such as confusion, fatigue, headache, and anosmia, but also fatal central nervous system (CNS) diseases such as ischemic stroke, meningoencephalitis, and encephalopathy have been reported (4–7).

Innate immune cells such as monocytes, macrophages, and microglia, the resident immune cells in the brain, serve as the first line of host defense against pathogens, including SARS-CoV-2. While macrophages are found in essentially all tissues where they patrol for potential pathogens and play a fundamental role in the inflammatory processes evolving from SARS-CoV-2 infection (8), microglia represent the resident immune cells in the brain and are key factors in COVID-19-related CNS disorders (9, 10). Studies focusing on the interaction between SARS-CoV-2 and human microglia are limited so far. However, recent post-mortem analysis of COVID-19 patients revealed the involvement of microglia leading to profound neuroinflammation and gliosis (11). Both macrophages and microglia express Toll-like receptors (TLR), including the single-stranded RNA (ssRNA)-sensing TLR7 and TLR8 localized to the endosomal compartment, as crucial components in the initiation of an immune response combating viruses, including SARS-CoV-2 (12). In principle, binding of viral ssRNA leads to dimerization and activation of the receptors with subsequent induction of a complex intracellular signaling pathway, which results in the activation of transcription factors, such as NF-κB and interferon regulatory factors (IRFs). In turn, immune cells release inflammatory molecules including cytokines (e.g. tumor necrosis factor alpha, TNF; interleukin (IL)-6), chemokines, and type I and II interferons (IFNs) (13, 14). In a subset of COVID-19 patients with an altered blood-brain-barrier, increased levels of specific cytokines, such as IL-6, in the cerebrospinal fluid (CSF) were detected (15). Rare, mostly loss-of-function mutations of TLR7, but also TLR3, another member of the TLR family, which detects double-stranded RNA, are present in up to 5% of severe COVID-19 cases under the age of 60 (16–18).

The SARS-CoV-2 reproduction cycle requires the transcription of the about 30 kB positive-sense RNA genome in which non-coding and translated RNA regions are transcribed to a varying extent depending on their genomic position (19). The SARS-CoV-2 genome harbors nucleotide motifs such as uracil (U)UU cytosine (C) and guanine (G)U that are capable of binding to the ssRNA-sensing sites of human (h)TLR7 and hTLR8, respectively, thereby mediating an inflammatory response (20). Cellular processes involving endogenous endo(ribo)nuclease activity, endocytosis, and cell death can lead to the generation of SARS-CoV-2 RNA fragments within the endosomal compartment (21, 22). In accordance with this, two SARS-CoV-2-derived sub-sequences were recently described to induce TLR7/8 signaling in human dendritic cells (23).

Here, we sought to systematically screen for SARS-CoV-2-derived RNA fragments that activate ssRNA-sensing TLRs. To this end, we made use of the *BrainDead* program, a recently developed machine learning approach that predicts small ssRNAs as potential ligands for TLR7/8 (24). First, the SARS-CoV-2 genome was fragmented into overlapping 22 nucleotide (nt) sub-sequences whose potential to activate TLR7/8 was then calculated by *BrainDead.* Out of almost 30,000 analyzed RNA fragments 32 sub-sequences were predicted as TLR7/8 activators that (i) are not preserved in the human translatome, (ii) are unlikely to form local base pairing within the SARS-CoV-2 genome, and (iii) are most likely single-stranded. In a second step, we employed a HEK hTLR7/8 reporter system to analyze and validate several top-ranked SARS-CoV-2 RNA fragments selected out of the 32 predicted sub-sequences as activators of hTLR7 and/or hTLR8. The functional relevance of these RNA fragments derived from different genomic regions of SARS-CoV-2 for driving an immune response in human was demonstrated by testing human macrophages differentiated from THP-1 monocytes and induced pluripotent stem cell (iPSC)-derived human microglia. Human macrophages and microglia incubated with the SARS-CoV-2 ssRNAs identified as novel hTLR7/8 activators released cytokines such as TNF and IL-6, respectively, in a sequence-dependent manner. Our data determine distinct RNA fragments derived from the SARS-CoV-2 genome as novel ligands for hTLR7/8, being able to activate both human peripheral and CNS immune cells.

## Materials and methods

### Filters for RNA fragment selection

The SARS-CoV-2 reference genome NC_045512.2 was fragmented using a sliding window approach of size 22 and a step size of 1. Fragments *present in human transcripts* (GRCh38) were identified using *BLAST* (*word_size 7, gapopen 5, penalty -3, reward 1*, as suggested for short sequences by NCBI). To ensure *BLAST* hits are within transcribed regions instead of intergenic sites, we cross-referenced the hits with a human reference genome GTF file and filtered them for sites intersecting annotated transcripts. RNAs with a *BrainDead* score of 0.85 or higher are considered to have a *high activation potential.* The threshold was selected based on the score histogram shown in **Figure S1**. *BrainDead* v1.0 with the final model from (24) was used. This model employs the microglia activation dataset available on its GitHub page, a Support Vector Machine with a radial basis function kernel and the k-mers AA, AGA, AGGU, AGU, AGUU, CU, GAA, GAGG, GG, GGG, GU, GUU, UGA, UGU, UU, UUG, UUGU, and UUU. For each fragment, its unpaired probability with respect to its genomic context was assessed *via* the ViennaRNA Python API *(RNAplfold v2.4.18, W=70, L=70, u=22*) (25). The base pairing potential of each fragment within the whole genome was investigated using *IntaRNA* (*v3.2.0, intLenMax=22, seedbp=4, helixMinBP=4, model=B, acc=N, i.e. excluding accessibility consideration)* (26, 27). Position, interaction length, and hybridization energy of the most stable (lowest energy) interaction was identified and used for filtering. An interaction was considered ‘local’ if the genomic distance between fragment and target was <= 2000 nt.

### Reagents

Resiquimod (R848) and loxoribine were purchased from *In vivo*Gen (San Diego, CA, USA). Lipopolysaccharide (LPS) was provided by Enzo Life Sciences (#ALX-581-012, Lörrach, Germany). TNF was acquired from Peprotech (#315-01A, Cranbury, NJ, USA). SARS-CoV-2 RNA fragments and control oligoribonucleotide, as indicated in the Figures and **Table 1**, were synthesized with 5′ phosphorylation and phosphorothioate bonds in every base (Integrated DNA Technologies, Coralville, IA, USA). Sequence information for all RNA fragments is provided in **Table 1**.

**Table 1:** List of 32 genomic fragments of 22 nt length that passed the filtering steps, and the two additional sequences s_15723 and s_20487 previously published (see text).

id	fragment	length	start	gene	score	Pr(unpaired)	mfe	mfe_len	mfe_dist
s_1886	GCUCGUGUUGUACGAUCAAUUU	22	1886	ORF1	0.89	0.000519	-18.3	12	3772
s_1887	CUCGUGUUGUACGAUCAAUUUU	22	1887	ORF1	0.88	0.000644	-17.7	13	10631
s_1888	UCGUGUUGUACGAUCAAUUUUC	22	1888	ORF1	0.87	0.00105	-17.7	13	10630
**s_1889**	**CGUGUUGUACGAUCAAUUUUCU**	**22**	**1889**	**ORF1**	**0.9**	**0.001334**	**-17.7**	**13**	**10629**
s_1890	GUGUUGUACGAUCAAUUUUCUC	22	1890	ORF1	0.88	0.005519	-17.2	13	10628
**s_1891**	**UGUUGUACGAUCAAUUUUCUCC**	**22**	**1891**	**ORF1**	**0.9**	**0.010529**	**-16.3**	**12**	**10627**
**s_1892**	**GUUGUACGAUCAAUUUUCUCCC**	**22**	**1892**	**ORF1**	**0.9**	**0.013507**	**-15.8**	**11**	**10626**
s_2032	UUUGGCUACUAACAAUCUAGUU	22	2032	ORF1	0.87	0.000835	-17.2	14	9767
**s_6746**	**UUAAACCGUGUUUGUACUAAUU**	**22**	**6746**	**ORF1**	**0.91**	**6.55E-05**	**-17.7**	**14**	**19305**
**s_6747**	**UAAACCGUGUUUGUACUAAUUA**	**22**	**6747**	**ORF1**	**0.91**	**6.54E-05**	**-17.7**	**14**	**19304**
s_9104	AGUUUACGCCCUGACACACGUU	22	9104	ORF1	0.85	0.001203	-17.8	13	4846
**s_9109**	**ACGCCCUGACACACGUUAUGUG**	**22**	**9109**	**ORF1**	**0.9**	**0.00013**	**-17.4**	**15**	**17965**
s_10142	CUUUGGCUUGAUGACGUAGUUU	22	10142	ORF1	0.86	0.000276	-18.7	16	11111
s_10143	UUUGGCUUGAUGACGUAGUUUA	22	10143	ORF1	0.86	0.000196	-16.5	15	7249
**s_11402**	**AAUGUCUUGACACUCGUUUAUA**	**22**	**11402**	**ORF1**	**0.9**	**0.011082**	**-18.1**	**14**	**9117**
s_11403	AUGUCUUGACACUCGUUUAUAA	22	11403	ORF1	0.89	0.019687	-17.2	13	9118
s_11404	UGUCUUGACACUCGUUUAUAAA	22	11404	ORF1	0.87	0.003992	-16.6	12	9119
s_11655	UUUGUUUACUCAACCGCUACUU	22	11655	ORF1	0.86	0.133579	-16.8	16	16903
s_11656	UUGUUUACUCAACCGCUACUUU	22	11656	ORF1	0.86	0.118832	-16.8	16	16902
s_14401	UUCCCACCUACAAGUUUUGGAC	22	14401	ORF1	0.85	0.000304	-15.5	16	10286
s_14402	UCCCACCUACAAGUUUUGGACC	22	14402	ORF1	0.85	0.000658	-16.3	13	4234
s_14403	CCCACCUACAAGUUUUGGACCA	22	14403	ORF1	0.85	0.000544	-17.2	9	14703
**s_15723**	**UGCUGUUGUGUGUUU**	**15**	**15723**	**ORF1**	**0.85**	**0.011121**	**-18.2**	**14**	**11769**
s_16363	UUGUCUGUUAAUCCGUAUGUUU	22	16363	ORF1	0.86	0.005162	-17.5	14	6831
s_16778	UCUUUACUGGUUAUCGUGUAAC	22	16778	ORF1	0.85	0.000702	-16.9	15	15896
**s_20487**	**GUGUGUGUGUUCUGUUAUUG**	**20**	**20487**	**ORF1**	**0.74**	**0.000023**	**-19.2**	**17**	**19673**
s_22912	UUACCUGUAUAGAUUGUUUAGG	22	22912	S	0.85	0.000718	-15.9	12	6686
**s_24432**	**AAGCUUUAAACACGCUUGUUAA**	**22**	**24432**	**S**	**0.9**	**0.00124**	**-18.2**	**13**	**3644**
s_25466	CUUCAGAUUUUGUUCGCGCUAC	22	25466	ORF3	0.89	0.000162	-16.7	17	24678
s_25467	UUCAGAUUUUGUUCGCGCUACU	22	25467	ORF3	0.86	0.000265	-16.7	12	2909
s_28222	AGUAUCAUGACGUUCGUGUUGU	22	28222	ORF8	0.88	0.000556	-16.6	16	19105
**s_28225**	**AUCAUGACGUUCGUGUUGUUUU**	**22**	**28225**	**ORF8**	**0.92**	**6.74E-05**	**-15.9**	**17**	**23677**
s_29574	UUUUCGCUUUUCCGUUUACGAU	22	29574	ORF10	0.85	0.001079	-18.4	17	2638
s_29575	UUUCGCUUUUCCGUUUACGAUA	22	29575	ORF10	0.85	0.000945	-18.4	17	2639

“start” denotes the first position of the fragment within the genomic sequence and defines the order of the table, as well as the sequence “id”. Sequences finally validated experimentally (score ≥ 0.9) are highlighted. “score” denotes the *BrainDead* prediction score with respect to murine microglia activation, while “Pr(unpaired)” indicates the probability that the whole fragment is unpaired within its genomic context. “mfe” denotes the minimum free (hybridization) energy of any RNA-RNA interaction between the fragment and a genomic region that can be predicted by IntaRNA. “mfe_len” and “mfe_dist” denote the respective interaction length and distance to target.

### Mice and cell lines

C57BL/6 mice were obtained from the FEM, Charité – Universitätsmedizin Berlin, Germany. Animals were maintained and handled in accordance with the German Animal Protection Law and approved by the Regional Office for Health and Social Services in Berlin (*Landesamt für Gesundheit und Soziales* – *LAGeSo*, Berlin, Germany). HEKBlue™ cells expressing mouse TLR7, human TLR7, or human TLR8, as well as the respective control cell lines HEK-Blue™ *Null2-k*, *Null1-k*, and *Null1* (*InvivoGen*, San Diego, CA, USA) were cultured in Dulbecco’s modified Eagle’s medium (DMEM; Invitrogen #41,965,062, Carlsbad, CA, USA). DMEM was supplemented with 10% heat-inactivated FCS (Gibco #10,082-147, Thermo Fisher Scientific, Waltham, MA, USA), penicillin (100 U/ml)/streptomycin (100 μg/ml; Gibco #15,140-122, Thermo Fisher Scientific, Waltham, MA, USA) and selection antibiotics Zeocin (100 µg/ml; *InvivoGen* #ant-zn, San Diego, CA, USA) and/or Blasticidin (10-30 µg/ml; *InvivoGen* #ant-bl, San Diego, CA, USA). THP-1 cells (provided by Dr. Elisabeth Kowenz-Leutz, Max Delbrück Center for Molecular Medicine, Berlin, Germany) were cultured in RPMI-1640 medium (Gibco #A1451701, Thermo Fisher Scientific, Waltham, MA, USA), supplemented with 0.05 mM 2-mercaptoethanol (Sigma-Aldrich #6250, Saint Louis, MO, USA), 10% heat-inactivated FCS, and penicillin (100 U/ml)/streptomycin (100 μg/ml). Cells were cultured at 37°C in humidified air with 5% (v/v) CO2.

### Primary cultures of murine microglia

Primary cell cultures of murine microglia were generated as previously described (28). Briefly, microglia were isolated from mouse brains on postnatal day (P). Meninges, superficial blood vessels, hippocampus, and cerebellum were removed from cortices. Cortices were then homogenized with 3 ml Trypsin (2.5%; Gibco #15090–046, Thermo Fisher Scientific, Waltham, MA, USA) for 25 min at 37°C. Trypsin reaction was stopped with 4 ml FCS, and 100 μl DNase (Roche #ROD 1284932, Basel, Switzerland) was added for 1 min. Cell suspension was centrifuged at 1200 rpm for 5 min. Pellets were resuspended in DMEM supplemented with 10% FCS and penicillin (100 U/ml)/streptomycin (100 μg/ml), mechanically dissociated, and passed through a 70 μm-cell strainer. Microglia were grown in PDL-coated T75 flasks (Sigma-Aldrich #P0899, Saint Louis, MO, USA) for 10-14 d in 12 ml DMEM at 37°C in humidified air with 5% (v/v) CO_2_. Microglial cells were separated from the underlying glial layer by shaking of the flasks for 20 min (300 rpm). Finally, 30.000 microglia/well were seeded in 96-well-plates in DMEM with 10% FCS and penicillin (100 U/ml)/streptomycin (100 μg/ml). On the following day, cells were used for experiments.

### Differentiation of iPSCs to CD43^+^ HPCs

The differentiation of iPSCs to primitive hematopoietic progenitor cells (HPCs) was performed as described previously (29). In brief, human induced pluripotent stem cells (iPSC BIHi-268-A10) were expanded in TeSR-E8 media (STEMCELL technologies #05990, Vancouver, BC, Canada) until they reached 80% confluency. Afterwards, cells were passaged with ReLeSR (STEMCELL technologies #100-0484, Vancouver, BC, Canada) into TeSR-E8 medium with 0.5µM Thiazovivin (STEMCELL technologies #72252 Vancouver, BC, Canada) onto Geltrex-coated (0.15 mg/ml) 6-well plates (ThermoFisher #A14133-02, Waltham, MA, USA). After 24 h, when approximately two 100 cell colonies per cm^2^ have been achieved, TeSR-E8 medium was replaced with 2 ml per well STEMdiff™ Hematopoietic Basal Medium plus Supplement A (1:200 dilution; STEMCELL technologies #05310, Vancouver, BC, Canada). After 48 h, half media change was performed. On day 3 complete media was exchanged by 2 ml/well of STEMdiff™ Hematopoietic Basal Medium plus supplement B (1:200 dilution; STEMCELL technologies #05310, Vancouver, BC, Canada). Without media removal, supplementation with 1 ml/well of medium B was performed on days 5, 7, and 9. On day 12, non-adherent cells were collected and centrifuged at 300xG for 5 min. FACS analysis has previously shown that these non-adherent cells represent highly pure populations (> 93%) of CD43^+^ hematopoietic progenitor cells (29).

### Differentiation of CD43^+^ HPCs to iPSC microglia

iPSC human microglia were differentiated from CD43^+^ HPCs, as previously described (30). In detail, HPCs were plated at a density of 130,000 cells per well onto 1 mg/ml Geltrex-coated 6-well plates in DMEM/F12 (ThermoFisher #11039021, Waltham, MA, USA) supplemented with 2X insulin-transferrin-selenite (ThermoFisher #41400045, Waltham, MA, USA), 2X B27 (ThermoFisher #17504001, Waltham, MA, USA), 0.5X N2 (ThermoFisher #17502048, Waltham, MA, USA), 1X glutamax (ThermoFisher #35050038, Waltham, MA, USA), 1X non-essential amino acids (ThermoFisher #11140035, Waltham, MA, USA), 400 μM monothioglycerol (Sigma-Aldrich #M1753, Saint Louis, MO, USA), and 5 μg/ml insulin (ThermoFisher #12585014, Waltham, MA, USA). Immediately before use, medium was supplemented with 100 ng/ml IL-34 (Peprotech #200-34, Cranbury, NJ, USA), 50 ng/ml TGFβ1 (Peprotech #100-21C, Cranbury, NJ, USA), and 25 ng/ml M-CSF (Peprotech #300-25, Cranbury, NJ, USA) taken from single-use frozen aliquots. On days 2, 4, 6, 8, and 10, 1 ml fresh media plus freshly thawed cytokine cocktail was added. On day 12, non-adherent cells were collected and centrifuged for 5 min at 300xG. The cell pellet was resuspended in 1 ml fresh medium plus tri-cytokine cocktail per well and added back to the same well. Media supplementation was continued (1 ml) on days 14, 16, 18, 20, 22, and 24. On day 25, non-adherent cells were centrifuged as on day 12. Cells were resuspended in 1 ml media/well plus 100 ng/ml IL-34, 50 ng/ml TGFβ1, 25 ng/ml M-CSF, 100 ng/ml CD200 (Bon-Opus, #C311, Millburn, NJ, USA) and 100 ng/ml CX3CL1 (Peprotech #300-31, Cranbury, NJ, USA) and added back to the same well. On day 27, 1 ml microglia media with five-cytokine cocktail was added per well. On day 28 cells were collected and plated in a 96-well plate (50.000 cells/well). On the following day, cells were used for experiments.

### Macrophage differentiation from THP-1 cells

Five days before use, THP-1 cells were seeded in cell culture medium in a 96-well plate (40.000 cells/well). For differentiation into macrophages cells were incubated with 100 ng/ml phorbol 12-myristate 13-acetate (PMA; Merck #P8139, Darmstadt, Germany) for 3 d. Cells were then washed twice with pre-warmed PBS, pH 7,4 (Gibco #10010023, Thermo Fisher Scientific, Waltham, MA, USA), and cultured for another 2 d in PMA-free medium before using for experiments.

### Enzyme-linked immunoabsorbent assay

Primary cultured mouse microglia, THP-1-derived macrophages, and human iPSC-derived microglia cells were incubated with 10 µg/ml of SARS-CoV-2 RNA fragments or control oligonucleotide complexed to LyoVec (*InvivoGen* #LYEC-RNA, San Diego, CA, USA) for 24 h. Subsequently, supernatants were collected and stored at -80°C. TNF and IL-6 concentrations in the supernatants were measured by Enzyme-Linked Immunoabsorbent Assay (ELISA) according to the manufacturer’s instruction (TNF alpha Mouse Uncoated ELISA Kit, Invitrogen, #88–7324-88, Carlsbad, CA, USA; TNF alpha Human Uncoated ELISA Kit, Invitrogen, #88–7346-88, Carlsbad, CA, USA; IL-6 Human Uncoated ELISA Kit, Invitrogen, #88–7066-88, Carlsbad, CA, USA).

### HEK-Blue TLR activation reporter assay

HEKBlue cells were seeded into 96-well plates (50.000 cells/well). After 24 h, the respective cell culture media was replaced by HEK-Blue Detection reagent (*InvivoGen* #hb-det2, San Diego, CA, USA), which was prepared according to the manufacturer’s instructions. Next, cells were incubated with 10 µg/ml of SARS-CoV-2 RNA-fragments or control oligonucleotide complexed to LyoVec for 24 h. The NF-κB/AP-1-inducible SEAP (secreted embryonic alkaline phosphatase) reporter protein was detected using the SpectraMax iD3 (Molecular Devices, San Jose, CA, USA) at a wavelength of OD 655 nm.

## Results

### Identification of SARS-CoV-2 RNA fragments with a high potential for TLR7/8 activation

**Figure 1:**
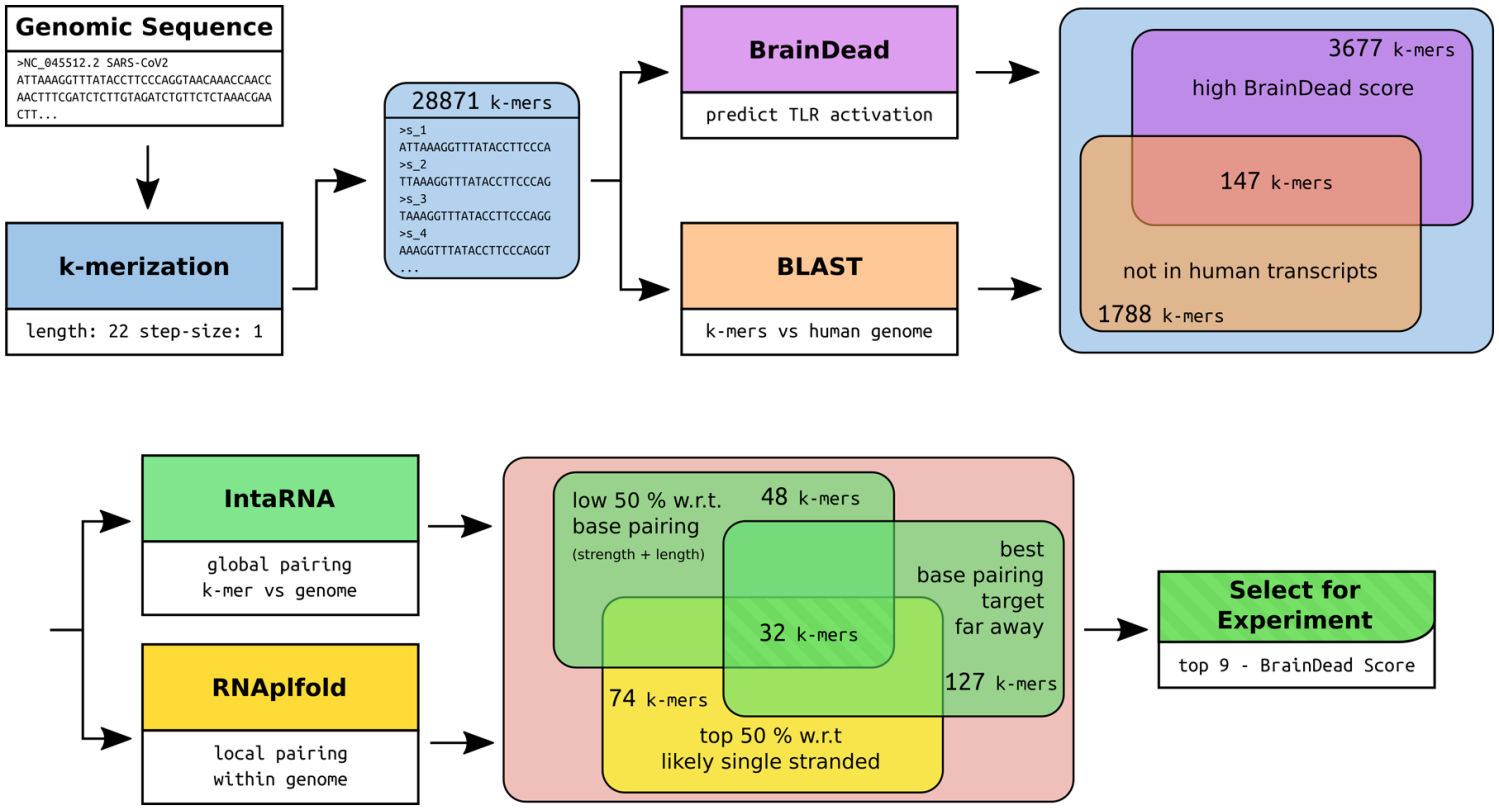
Workflow of the RNA fragment selection with respective sizes of the filtered subsets of interest and the tools used for filtering and subset-feature calculation. The potential for TLR7/8 activation was predicted using *BrainDead*. Sequences not found in human transcripts being predicted with high activation potential were selected (*BrainDead* score of 0.85 or higher, see *Methods and materials*). Subsequently, we focused on sequences that are free of strong base-pairing, which were determined by considering two quantities. One is being free of internal base-pairs, which is determined by single-strandedness in terms of unpaired probabilities (*RNAplfold*), and selecting the top 50% quantile (high single-strandedness). Similarly, RNA fragments were supposed to be free of base-pairing with other genomic sequences to increase the likelihood that the fragment can be bound by ssRNA-sensing TLRs. Again, the bottom 50% quantile with respect to the interaction strength (*IntaRNA*) was selected. To exclude local, possibly intramolecular structure formation, sub-sequences with close (<= 2000 nt) base pairing targets were excluded. Finally, the top 9 sequences with respect to their *BrainDead* score were selected for experimental validation.

Recently, we introduced *BrainDead*, a machine learning approach that predicts short ssRNAs with a high potential to act as TLR7/8 ligands based on sequential and structural features (24). Since the *BrainDead* prediction model was originally trained for microRNAs (miRNAs) acting as TLR ligands, and the exact nature and molecular mechanisms involved in SARS-CoV-2 fragmentation *in vivo* are unclear so far, we decided to analyze genome fragments with a length of 22 nt, being the average length of miRNA, as well as similar to established ssRNA agonists for TLR7/8 (13, 31, 32), providing distinct overlapping sub-sequences (**Figure 1**). Subsequently, 147 sub-sequences out of the 29,871 genomic RNA fragments, which (i) were not present in human transcripts (3,677 not found in transcripts of GRCh38) and (ii) were predicted by *BrainDead* to feature a high potential to activate TLR7/8 (1,788 with score ≥ 0.85), were filtered. The first filter step provided us with SARS-CoV-2-specific fragments not being part of the human host’s transcriptome, while the second step filtered for relevant candidates with respect to immune cell activation.

Since we focused on ssRNA-sensing TLRs in our study, we further pruned this data set based on three criteria: (i) high probability to be single-stranded, (ii) low/weak base pairing potential, and (iii) best base pairing target not being in local genomic proximity. We assessed a fragment’s potential to be single-stranded by its unpaired probability within its genomic context computed by RNAplfold. To avoid arbitrary probability thresholds, we focused on the top (highest) 50% with respect to unpaired probability. To further reduce the likelihood that a fragment is involved in base pairing rather than being single-stranded, we predicted the base pairing of each fragment within the whole genome using IntaRNA. Given that the minimum free energy, reported for each fragment, provides a measure of stability (the lower the more stable the interaction), we again focused on the top (least stable) 50% of all fragments. To further reduce the likelihood that a fragment is involved in local structure formation, we enforced that the IntaRNA-reported most table interaction site is located at least 2,000 nt down- or up-stream of the fragment’s position. This threshold was used to exclude fragments that possibly form intramolecular structures. The described filtering pipeline led to the identification of 32 candidate SARS-CoV-2 RNA fragments, from which we selected the top 9 candidates with respect to the *BrainDead*-predicted TLR7/8 activation potential (**Figure 1**). The final list of SARS-CoV-2 RNA fragments (**Table 1**) contained 4 ssRNAs (s_9109, s_11402, s_24432, s_28225) with unique location within the viral genome, while the two ssRNAs s_6746 and s_6747 at genomic position 6746 and 6747, respectively, overlap. The fragments s_1889, s_1891, and s_1892 overlap due to the systematic sequence-fragmentation and filtering procedure. Additionally, we included two GU-rich RNA fragments (**Table 1**) that have been recently described to induce hTLR7/8 signaling in dendritic cells (DCs) thereby triggering IFN release and an inflammatory response (23). These two RNA fragments, termed here as s_15723 (15 bases, *BrainDead* score 0.85) and s_20487 (20 bases, *BrainDead* score 0.74), frequently occur in human transcripts (s_15723/s_20487: 174/844 times, respectively).

### SARS-CoV-2 RNA fragments activate TLR7 and/or TLR8 in a sequence-dependent manner

The *BrainDead* machine learning program was originally developed and trained based on data derived from assays testing the production of cytokines, such as TNF, in mouse microglia in response to ssRNA (24). Thus, we analyzed the potential of primary mouse microglia, which express all known TLRs including TLR7 (32, 33), to respond to the synthesized candidate SARS-CoV-2 RNA fragments identified as potential TLR7/8 activators above. To this end, cells were incubated with the respective RNA fragment, and concentration levels of released TNF indicating microglial activation were assessed by Enzyme-linked Immunosorbent Assay (ELISA) after 24 h (**Figure S2**). The RNA fragments s_1889, s_6746, s_6747, s_11402, s_24432, and s_28225 significantly induced TNF release resulting in a validation rate of 67% (6 out of 9 fragments). The highest TNF concentrations in microglial supernatants were assessed after incubation of the cells with s_11402, s_1889 and s_28225. TNF concentration levels in supernatants of microglia incubated with s_6746 and s_6747, whose sequences only differ in two bases (see **Table 1**), were similar and not substantially different from the concentration levels measured in the supernatant of cells incubated with the s_24432 fragment. The fragments s_1891 and s_1892 did not induce significant TNF release, while the s_1889 fragment with a 20 nt/19 nt base overlap with fragments s_1892/s_1892 potently induced TNF. Incubation with the s_15723 ssRNA led to, although not reaching statistical significance, moderate TNF release, while the s_20487 fragment induced significant TNF production in murine microglia. In general, TNF responses induced by the significantly activating RNA fragments predicted by the *BrainDead* program were stronger compared to the tested RNA fragments previously described by Salvi and colleagues (23). Mouse microglia also responded to the TLR7 agonist loxoribine, the TLR7/8 agonist resiquimod (R848), and the TLR4 agonist lipopolysaccharide (LPS), as expected, while no TNF was detected in cell cultures incubated with a control oligonucleotide harboring a scrambled sequence (**Figure S2**) (32, 33). Next, we sought to assess the SARS-CoV-2 RNA fragments’ ability to specifically induce murine (m)TLR7 signaling. To this end, we employed HEK293 cells, stably co-expressing mTLR7 and an NF-κB/AP1-inducible secreted embryonic alkaline phosphatase (SEAP) reporter gene (**Figure S3**). The SARS-CoV-2-derived fragments s_1889, s_1891, s_9109, s_11402, s_24432, s_28225, s_15723, and s_20487 induced significant NF-κB activation in mTLR7 reporter cells, and this response was sequence-specific. The RNA fragments s_1892, s_6746, and s_6747 also induced NF-κB activation, although not reaching a statistically significant level (**Figure S3**). Overall, the response of mTLR7 reporter cells to the respective RNA fragment revealed a similar pattern with respect to the degree of activation as primary mouse microglia (see **Figures S2, S3**). In summary, the SARS-CoV-2-derived RNA fragments determined by *BrainDead* to activate TLR7/8 induced an inflammatory response driven by primary mouse microglia and induced mTLR7 signaling in a sequence-dependent fashion.

**Figure 2:**
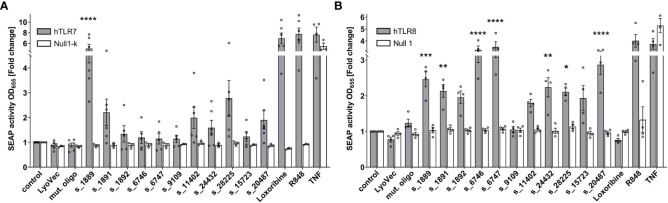
SARS-CoV-2 RNA fragments activate human TLR7 and/or human TLR8. HEK-Blue cells co-expressing human TLR7 **(A)** or human TLR8 **(B)** and an NF-κB/AP1-inducible secreted embryonic alkaline phosphatase (SEAP) reporter gene were incubated with selected SARS-CoV-2 RNA fragments (10 μg/ml), as indicated, for 24 h. Incubation with loxoribine (1 mM) and R848 (10 μg/ml) served as positive control for TLR7 and TLR7/8 activation, respectively. TNF (100 ng/ml) was used as positive control for SEAP induction. Incubation with mutant oligoribonucleotide (10 μg/ml), transfection agent alone (LyoVec), and unstimulated condition (control) served as negative control. HEK-Blue Null1-k and HEK-Blue Null1 cells served as negative cell control. Data are pooled from 4-6 independent experiments. Results are expressed as mean ± SEM. Data were analyzed by one-way ANOVA followed by Dunnett’s *post hoc* test. *p < 0.05, **p < 0.01, ***p < 0.001, ****p < 0.0001 *vs*. control.

To systematically investigate the ability of the predicted SARS-CoV-2 RNA fragments to induce human (h) TLR signaling and to distinguish between responses mediated by hTLR7 and/or the phylogenetically and structurally highly related hTLR8 (13, 34), we employed HEK293 cells stably co-expressing hTLR7 (**Figure 2A**) or hTLR8 (**Figure 2B**), as well as the NF-κB/AP1-inducible SEAP reporter gene. Among the tested SARS-CoV-2 RNA fragments s_1889, s_1891, s_11402, s_24432, s_28225, and s_20487 induced an increase in NF-κB activation in hTLR7 reporter cells, compared to control condition, although statistical significance was only achieved for the first fragment. While the fragments s_6746, s_6747, and s_15723 induced mTLR7 signaling (see **Figure S3**), hTLR7 reporter cells did not respond to them (**Figure 2A**). We have shown in previous studies that ssRNAs with a high *BrainDead* score feature a high probability to activate hTLR8 (24). In line with this, the SARS-CoV-2-derived RNA fragments s_1889, s_1891, s_6746, s_6747, s_24432, s_28225, and s_20487 significantly induced NF-κB activation in hTLR8 reporter cells. Similarly, although not reaching statistical significance, s_1892 and s_11402, as well as s_15723 induced hTLR8 activation. In contrast, the fragment s_9109 did not activate hTLR8 (**Figure 2B**). While the fragments s_6746 and s_6747 potently induced NF-κB activation in hTLR8 reporter cells, no such response was observed in reporter cells transfected with hTLR7 (**Figure 2A**). Overall, these results indicate a sequence- and species-specific recognition of SARS-CoV-2 ssRNA through hTLR8 in particular, but also hTLR7.

### RNA fragments derived from distinct SARS-CoV-2 genomic regions activate human macrophages

**Figure 3:**
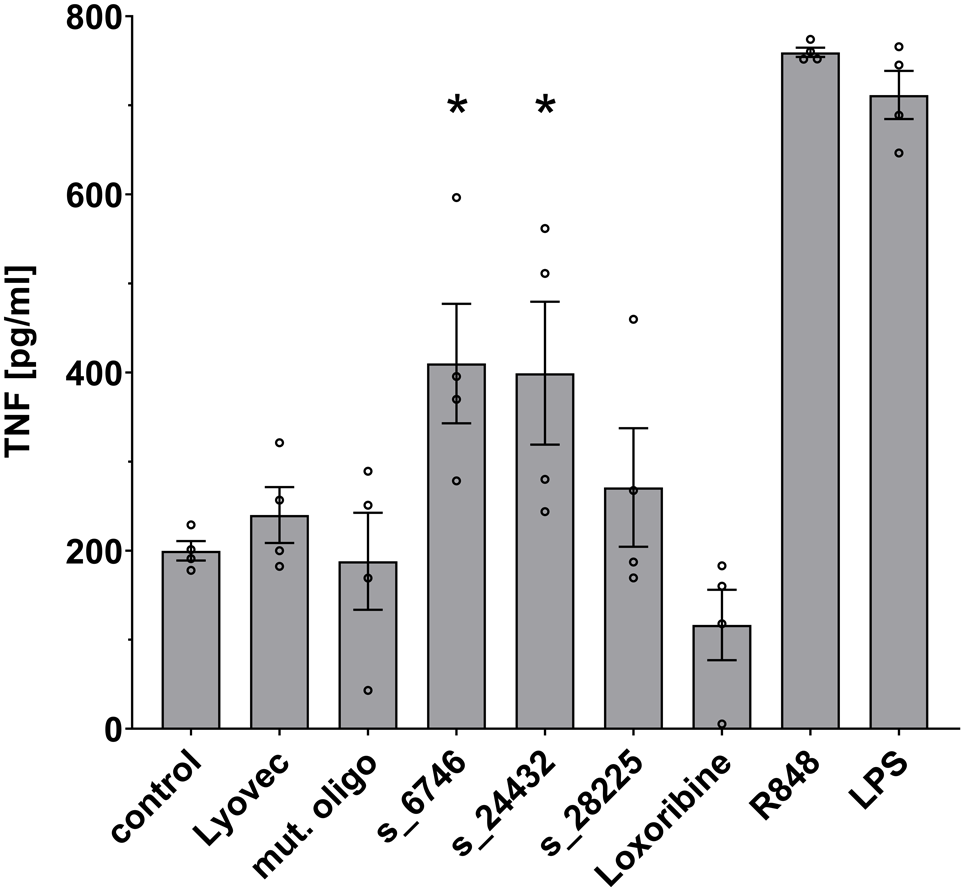
Human macrophages release TNF in response to specific SARS-CoV-2 RNA fragments. Human macrophages differentiated from THP-1 monocytes were incubated with various SARS-CoV-2 RNA fragments (10 μg/ml), as indicated, for 24 h. Subsequently, supernatants were analyzed by TNF ELISA. Untreated cells (control), mutant oligoribonucleotide (10 μg/ml), and the transfection agent LyoVec alone served as negative control. LPS (100 ng/ml) and R848 (10 μg/ml) served as positive control for TLR4 and TLR7/8 activation, respectively. Results are expressed as mean ± SEM. Data were analyzed by one-way ANOVA followed by Dunnett’s *post hoc* test. *p < 0.05 *vs*. control (*n* = 4).

We sought to investigate the potential of the SARS-CoV-2 RNA fragments identified as novel hTLR7 and/or hTLR8 ligands to activate human immune cells such as macrophages, which are essentially present in all organs, act as key innate immune cells, and play a crucial role in COVID-19-related inflammation (8). Taking into account RNA seq and ribo-seq data on SARS-CoV-2 RNA expression and translation during infection (19), we selected 3 out of the 9 candidate SARS-CoV-2 RNA fragments determined by *BrainDead* to activate TLR7/8 and being located to distinct SARS-CoV-2-derived genomic regions. In detail, we focused on s_6746 located to the open reading frame (ORF)-1, s_24432 to the spike protein-coding region, and s_28225 present in ORF-8. All three ssRNAs exhibited a high *BrainDead* activation score (see **Table 1**). To test their functional impact on human macrophages, macrophages differentiated from human THP-1-derived monocytes were incubated with the respective synthesized oligoribonucleotides. Subsequently, TNF amounts in the supernatants were determined by ELISA (**Figure 3**). The SARS-CoV-2 RNA fragments s_6746 and s_24432 significantly induced TNF release from human macrophages. Similarly, although not reaching statistical significance, supernatants of cells incubated with the fragment s_28225 contained elevated TNF amounts compared to control (**Figure 3**).

### RNA fragments from distinct SARS-CoV-2 genomic regions activate human microglia

**Figure 4:**
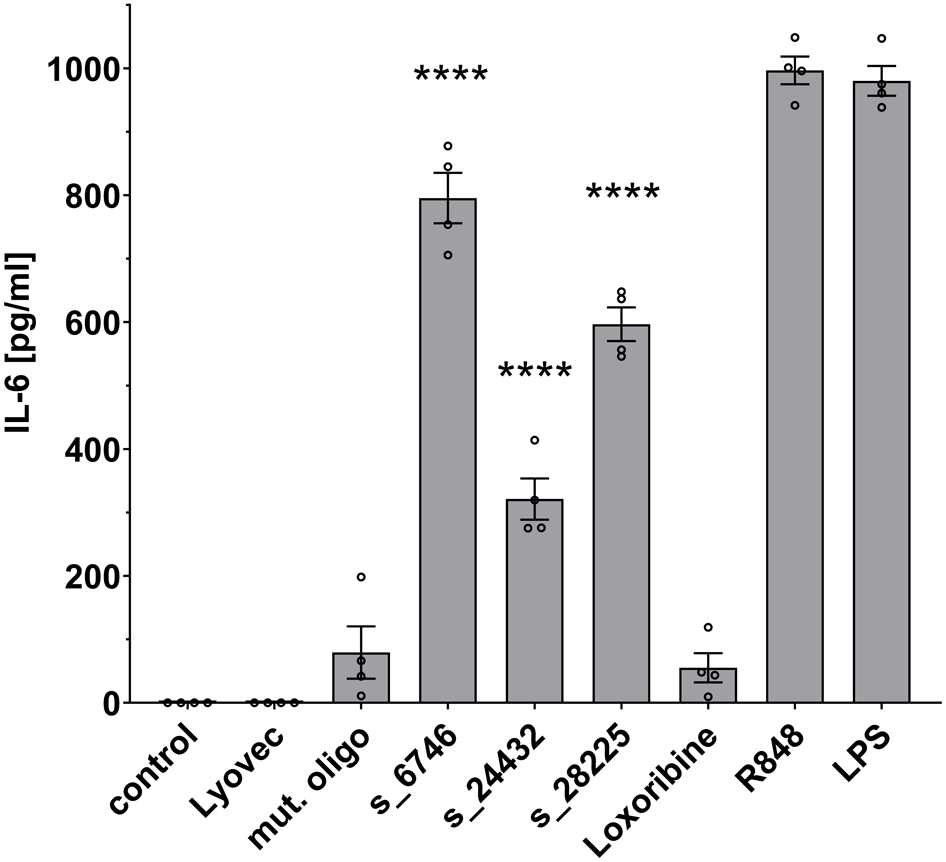
IPSC-derived human microglia (iMGL) release IL-6 in response to distinct SARS-CoV-2 RNA fragments. iMGL were incubated with selected SARS-CoV-2 RNA fragments (10 μg/ml), as indicated. Subsequently, supernatant was analyzed by IL-6 ELISA. Untreated cells (control), mutant oligoribonucleotide (10 μg/ml), and LyoVec served as negative control. LPS (1 μg/ml) and R848 (10 μg/ml) served as positive control. Results are expressed as mean ± SEM. Data were analyzed by one-way ANOVA followed by Dunnett’s *post hoc* test. ****p < 0.0001 *vs*. control (*n* = 4).

Microglia become activated in response to various stimuli, and transforming into an inflammatory state they release inflammatory molecules, such as TNF, IL-6, and IL-1β (35). To investigate the ability of the selected SARS-CoV-2 RNA fragments identified by *BrainDead* and validated as TLR7/8 ligands, to activate human microglia, we made use of human iPSC-derived microglia (iMGL), which have been recently characterized as a valid model system for the investigation of human microglia (30, 36). To this end, iMGL were exposed to s_6746, s_24432, s_28225, or the control mutant oligoribonucleotide. Subsequently, supernatants were analyzed for IL-6 amounts, as this cytokine is primarily released from iMGL activated by RNA-based TLR7/8 agonists (data not shown) and its concentration in the cerebrospinal fluid (CSF) of a subset of COVID-19 patients is increased (15) (**Figure 4**). All three tested SARS-CoV-2-derived RNA fragments significantly induced IL-6 release from iMGL. In particular, s_6746 induced the most potent response, followed by s_28225 and s_24432. Notably, the RNA fragment s_28225 strongly induced IL-6 production in iMGL, whereas TNF production in human macrophages in response to this RNA fragment was only moderate (see **Figures 3**, **4**). These findings suggest a cell type- and cytokine-specific mode of action on human immune cells triggered by SARS-CoV-2 RNA fragments.

In summary, our data show that RNA fragments from distinct SARS-CoV-2-derived genomic regions activate both human macrophages and microglia, thereby generating an inflammatory response.

## Discussion

Our study identifies distinct SARS-CoV-2 RNA fragments derived from different genomic locations as novel hTLR7 and/or hTLR8 ligands, which are capable of inducing cytokine release from human macrophages and microglia in a sequence-dependent fashion. SARS-CoV-2 is generated from a single-stranded positive-sense RNA genome of about 30 kB. However, not all parts of the SARS-CoV-2 genome are expressed and translated equally during infection. Particularly, RNA-seq, as well as Ribo-seq analyses of SARS-CoV-2-infected human lung cancer cells and epithelial kidney cells from *African green monkey* revealed an enhanced expression and translation rate at the 3’-end of SARS-CoV-2 (19). Whereas the top *BrainDead*-ranked SARS-CoV-2-derived RNA fragment s_28225 identified as hTLR8 ligand in our study is located near the genome’s 3’-end in ORF-8, which encodes a SARS-CoV-2-specific protein central for immune evasion (37), the fragment s_24432, also activating hTLR8, is located within the spike gene near the end of the SARS-CoV-2 genome. The other seven analyzed RNA fragments, as well as the comparatively tested SARS-CoV-2-derived oligoribonucleotides reported previously by Salvi et al. (23) locate to ORF-1, which represents the largest (about 75%) part of the SARS-CoV-2 genome. Still, the exact nature and resulting sequence pattern of endosomal genome fragmentation of SARS-CoV-2 *in vivo* is unknown. The endosomal RNAses 2 and T2 play a key role in nucleic acid degradation of various pathogens producing RNA fragments recognized by TLR8. While both RNases cleave the motifs UU, CU, and UC, RNAse 2 preferentially cuts before, and RNase T2 after U (21, 38, 39). Since the RNA fragments tested in our study derive from SARS-CoV-2 genomic regions with high probability of single-strandedness, low evidence for local base pairing, and more than 30% of the SARS-CoV-2 genome contain U (18), RNase 2 and T2 may well bind and operate within the proximity of the tested RNA fragments, thereby producing ssRNAs of various sizes sensed by TLR8, and probably also by TLR7. However, one limitation of our combined bioinformatic and experimental approach is that it does not confirm the presence and pathophysiological relevance of the tested SARS-CoV-2 RNA fragments *in vivo*, i.e. in COVID-19 patients. We suggest that endosomal processing of SARS-CoV-2 RNA can give rise to multiple fragments that are capable to trigger innate immune activation. Even if the 22 nt RNA fragments from SARS-CoV-2 identified as TLR7/8 activators in our study are length-wise not exactly generated *in vivo*, based on the fact that TLR7/8 recognizes single nucleosides, as well as di- and trinucleotides, the involved nucleases may be capable of cleaving SARS-CoV-2 RNA in such a way that the resulting fragments are recognized by the ssRNA-sensing immune receptors (40). This is in line with recent bioinformatic-based data (20) indicating that the SARS-CoV-2 genome harbors multiple fragments that are rich in GU motifs required for TLR7/8 binding (13, 31). Furthermore, as the tested SARS-CoV-2 fragment sizes allow only for minor structure formation, as it would be expected for strong fragmentation, and are representative for subsequences of longer fragments that are unpaired, the data presented in our study should extrapolate to at least some extent. To determine which exact SARS-CoV-2 RNA fragments are produced *in vivo* follow-up experiments should analyze tissue and fluid samples containing SARS-CoV-2-infected cells from COVID-19 patients. SARS-CoV-2 fragments/sequences resulting from endosomal degradation may vary depending on time (i.e. early versus late infection stages) and/or severity of the infection. Thus, future work is needed to unravel the details of endosomal genome fragmentation of SARS-CoV-2 and may shed light on the identity of SARS-CoV-2 RNA fragments and the abundance of transcription, as well as the relevance of the sequence patterns identified in our study *in vivo*.

TLR7 was recently reported to be activated by pattern molecules derived from coronavirus, and the subsequently triggered signaling pathway *via* IRF3/7 and NF-κB results in type I IFN, IL-1β, IL-6, and TNF expression in immune cells (41–43). However, the molecular details of the interaction between the ssRNA-recognizing TLRs and SARS-CoV-2 remained undetermined so far. In principle, TLR7 and TLR8 feature two different ligand-binding sites. At the respective ssRNA-binding site (also termed second binding site) specific motifs of at least three nucleotides are required for binding, whereas the first binding site interacts with single nucleosides, such as G and/or U (44, 45). These two binding sites can affect each other, as ssRNA binding to hTLR7 leads to an increased affinity for G at the first binding site (44, 46). Interaction between an oligoribonucleotide and the respective binding site results in homodimer formation and subsequent receptor activation (44, 46). While hTLR7 binds to ssRNA harboring U-rich motifs, such as UUUC, pre-formed hTLR8 dimers recognize single U and UUG at the respective binding site (45). UUU and UUC interact with the second binding site of hTLR7 with highest affinity, while UUG and UUA binding exhibits medium affinity. Of note, UUU, (UUU)_2_ and UUC were found more frequently in the SARS-CoV-2 genome than in the SARS-CoV genome (20). Our experimental data on hTLR7 activation by ssRNA derived from SARS-CoV-2 indicate that the presence of motifs with full or medium binding affinity within a SARS-CoV-2-derived RNA fragment *per se* is not sufficient to activate this receptor. In detail, the SARS-CoV-2 RNA fragments s_6746 and s_6747, both containing UUUG (UUU: full affinity; UUG: medium affinity) and UUA (medium affinity) motifs present at different positions, did not induce hTLR7 signaling. Instead, these fragments induced strong activation of hTLR8 (see **Figure 2**). Comparative analysis of the s_1889, s_1891, and s_1892 fragments, which all harbor UUG (medium affinity) and UUUUC (two overlapping high affinity motifs: UUU, UUC) motifs required for hTLR7 activation, suggests that minimal variation of sequence and position of full and medium affinity motifs affect/modulate receptor activation. The RNA fragments s_15723 (GU ratio 93%) and s_20487 (GU ratio 85%) recently reported to induce TLR7/8 signaling in human DCs (23) and comparatively tested in our study, activated hTLR8, as expected. However, compared to these fragments the GU-low SARS-CoV-2-derived fragments s_6746 (GU ratio 59%) and s_6747 (GU ratio 55%) identified by *BrainDead* induced a more potent hTLR8 activation. This is consistent with previous findings that neither GU content nor the presence of uridine (ssRNA41, rRNA Sa12) is the only sequence features relevant for TLR8 activation (47). Collectively, our study shows that i) specific nucleotide motifs such as UUUC are not sufficient to activate hTLR7 and ii) hTLR8 activation does not exclusively depend on the GU-content of a given ssRNA. In line with this, not only GU-rich SARS-CoV-2 RNA fragments, as it has been previously shown (23), are able to activate human immune cells, but also fragments harboring varying sequence motifs from different locations within the viral genome. These SARS-CoV-2-derived ssRNAs differing in sequence motifs induce inflammatory responses, i.e. cytokine release, to varying degrees. Thus, interaction between TLR7 and TLR8 and their different SARS-CoV-2-derived ssRNA ligands may fine tune and orchestrate the human immune response against the virus. In addition to the selected SARS-CoV-2-derived RNA fragments predicted by *BrainDead* and experimentally confirmed as TLR7/8 ligands activating human immune cells, our study presents 23 further sequences derived from the SARS-CoV-2 genome whose relevance for triggering an immune response through TLR signaling may be addressed in future studies.

Besides the attachment to the host angiotensin enzyme 2 (ACE2) receptor, the priming of S protein by transmembrane protease serine subtype 2 (TMPRSS2) and subsequent membrane fusion, as well as the cathepsin-dependent endosomal uptake used by viruses to infect cells lacking TMPRSS2 and resulting in TLR activation, are suggested to be key for the entry of SARS-CoV-2 into cells (48–50). While previous variants of SARS-CoV-2 predominantly utilize membrane fusion, the Omicron variant favors endosomal uptake, leading to a wider range of potential target cells and pointing to a relevance of TLR signaling for the elimination of the virus (51, 52). Apart from being essential for viral entry, lysosomes containing TLR7/8 appear to be involved in the main exit pathway from infected cells (53, 54). SARS-CoV-2 infection of the brain results in neuroinflammation featuring axonal damage, perivascular inflammation, and microgliosis (11, 55–58). Especially, SARS-CoV-2 infection of the brain leads to a local CNS response mediated through microglia driving neuroinflammation. This innate immune response correlates with alterations in the CSF, where amounts of inflammatory mediators such as IL-6 are increased (3, 15). We demonstrated in previous studies that the exposure of TLR7 and TLR8 in the CNS to both extracellular endogenous and viral ssRNAs containing specific sequence motifs can trigger neuroinflammatory and neurodegenerative processes (32, 33, 59, 60). Thus, it is conceivable that the SARS-CoV-2 RNA fragments analyzed in our current study, sharing some of the sequence motifs that are responsible for RNA detection through TLRs in microglia (32, 59), are also capable of inducing CNS damage in COVID-19 patients. It has been recently shown that human microglia express ACE2 and TMPRSS2 (61). As these factors allow SARS-CoV-2 to infect cells, we sought to analyze the potential of distinct SARS-CoV-2 RNA fragments to activate human microglia, which express both TLR7 and TLR8 (62). All tested SARS-CoV-2 RNA fragments, as well as the TLR7/8 agonist R848 induced IL-6 release, while the TLR7-specific agonist loxoribine did not. In accordance with this, SARS-CoV-2 was recently shown to infect the human microglial cell line human microglial clone 3 (HMC3), resulting in the release of various cytokines, including IL-6 (63). Most of the SARS-CoV-2 RNA fragments analyzed in our study exclusively activated hTLR8 (see **Figure 2**), indicating that TLR8 serves as the predominant SARS-CoV-2 RNA-sensing receptor in human microglia. Of note, most of the tested SARS-CoV-2 RNA fragments did not only activate human, but also mouse immune cells, namely primary mouse microglia, which express both TLR7 and TLR8 (12). In accordance with this, these RNA fragments induced NF-κB signaling in mTLR7 reporter cells. Also, SARS coronavirus was recently shown to induce cytokine secretion from the murine macrophage line RAW264.7 through TLR7/8 (64).

Our data show that human macrophages significantly release TNF in response to the SARS-CoV-2 RNA fragments s_6746 and s_24432 (see **Figure 3**) that are capable of activating hTLR8, but not hTLR7, as shown in HEK TLR7/8 reporter cells (see **Figure 2**). Consistently, human macrophages respond to the TLR7/8 agonist R848, but not to the TLR7 agonist loxoribine. Overall, these findings imply that the specific SARS-CoV-2 fragments activate human macrophages through hTLR8, which is, like TLR7 expressed in these cells (65–68). Similarly to our findings in human macrophages, human microglia were activated by the SARS-CoV-2 RNA fragments s_6746 and s_24432 (see **Figure 4**). Likewise, the fragment s_28225, which induced an hTLR8 and a milder, statistically not significant hTLR7 response, activated these cells (see **Figures 2** and **4**). Furthermore, human microglia responded not only to the RNA fragments mentioned above, but also to R848, but not to loxoribine. These findings suggest that the tested SARS-CoV-2 fragments activate human microglia particularly through hTLR8, which was like TLR7, previously shown being expressed in human iPSC-derived microglia (36). Taken together, most of the tested SARS-CoV-2 RNA fragments that activated human immune cells in our study induced hTLR8 signaling, implying the receptor’s crucial role in the COVID-19-related inflammatory state. Still, the *BrainDead* top-ranked s_1889 sequence additionally induced a potent response in hTLR7 reporter cells (see **Figure 2**). Accordingly, the hTLR7-selective agonist imiquimod, an imidazoquinoline derivative, triggers an immune response that counters SARS-CoV-2 infection in its early phase (69). Yet, the detailed impact of specific hTLR7 or hTLR8 agonists, as well as dual TLR7/8 agonists such as R848 (70), on SARS-CoV-2 infection remains elusive and requires further clinical investigation considering different immune cell types and infection stages. We believe that the identification of SARS-CoV-2 RNA fragments as novel TLR7/8 activators will help i) to understand the mechanisms how SARS-CoV-2 activates human immune cells, i.e. innate immune activation can be induced by virus-derived genome fragments, and ii) to decipher the specific sequence motifs within such RNA fragments required for the activation of TLR7/8. Our data could serve as a basis for future studies leading to the design of new therapeutic options, e.g. the generation and/or optimization of novel sequence-tailored TLR7/8 agonists and/or antagonists, and further targeted immunomodulation-based therapies for COVID-19 patients (69, 71, 72).

In summary, our study indicates a role of both TLR7 and TLR8 in the SARS-CoV-2-triggered immune response, depending on the distinct sequence of the viral RNA fragment acting as their respective ligand. Thereby, our data expand previous findings on the detection of SARS-CoV-2 by nucleic acid-sensing immune receptors and provide further insight into the molecular mechanisms how this virus may trigger and modulate inflammation in COVID-19 patients.

## Data availability statement

The original contributions presented in the study are included in the article/**Supplementary Material**. Further inquiries can be directed to the corresponding authors.

## Ethics statement

The animal study was reviewed and approved by Landesamt für Gesundheit und Soziales Berlin.

## Author contributions

SL, RB, TW, and MR conceived the study and wrote the manuscript. TW, MR, DR, LH, MB, HW, and HK planned and carried out the experiments, and/or contributed to writing the manuscript. CK carried out the experiments. All authors contributed to the article and approved the submitted version.
