# Supplementary material for: Distinct SARS-CoV-2 RNA fragments activate Toll-like receptors 7 and 8 and induce cytokine release from human macrophages and microglia
Source: Front Immunol. 2023 Jan 13;13:1066456. doi: 10.3389/fimmu.2022.1066456 (PMC9880480; doi:10.3389/fimmu.2022.1066456)
Supplement: Supplementary file 1 [file DataSheet_1.pdf]

Supplementary Material

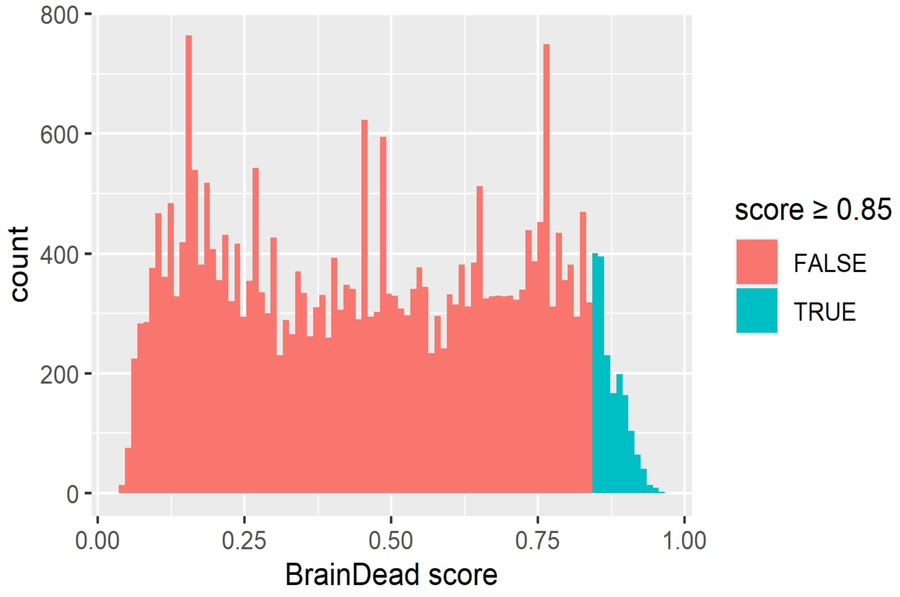

Figure S1. Distribution of *BrainDead* scores among the 29,871 genomic fragments of length 22 nt. Fragments considered to have a *high activation potential* are colored in blue featuring a score ≥ 0.85. This subset comprises 3,677 RNAs, i.e. the top-scored 8.1% of the data.

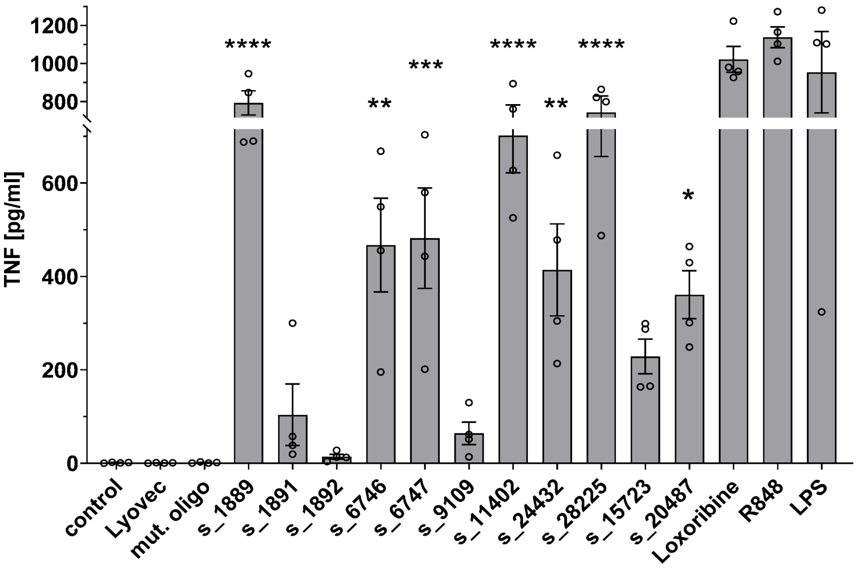

Figure S2. Selected SARS-CoV-2 RNA fragments determined as TLR7/8 ligands *in silico* activate primary mouse microglia. Microglia isolated from C57BL/6 mice were incubated with different synthetic SARS-CoV-2 RNA fragments (10 µg/ml), as indicated, for 24 h. Untreated cells (control), mutant oligoribonucleotide (10 µg/ml), and LyoVec served as negative control. LPS (100 ng/ml), Loxoribine (1 mM), and R848 (10 µg/ml) served as positive control. TNF amounts in the supernatant were assessed by ELISA. Results are expressed as mean±SEM. Data were analyzed by one-way ANOVA followed by Dunnett's post hoc test. *$p < 0.05$, **$p < 0.01$, ***$p < 0.001$, ****$p < 0.0001$ *vs*. control ($n = 4$).

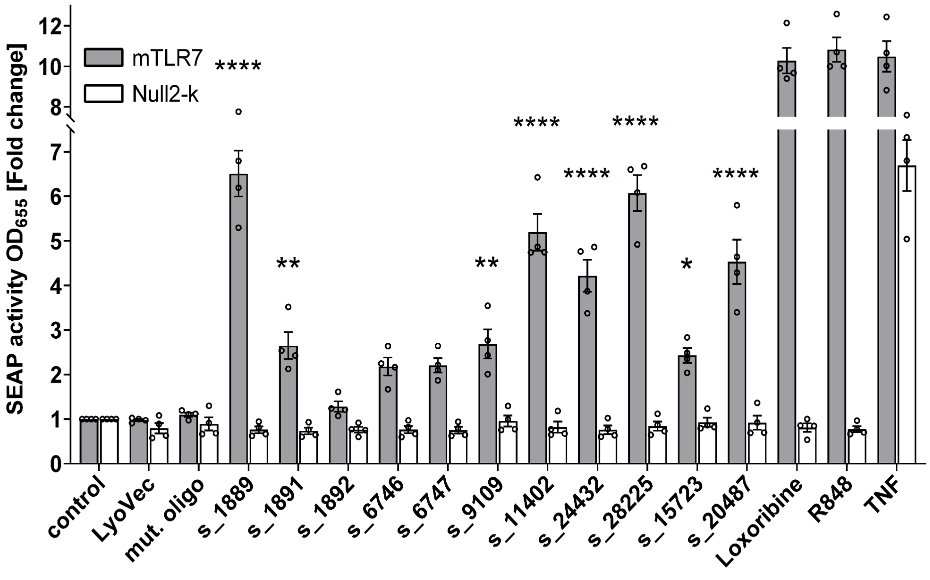

Figure S3. Selected SARS-CoV-2 RNA fragments determined as TLR7/8 ligands *in silico* induce murine TLR7 signaling. HEK-Blue cells coexpressing murine TLR7 and an NF-κB/AP1-inducible secreted embryonic alkaline phosphatase (SEAP) reporter gene were incubated with different synthetic SARS-CoV-2 RNA fragments (10 µg/ml), as indicated, for 24 h. Cells were also incubated with synthetic mutant oligoribonucleotide (10 µg/ml), transfection agent alone (LyoVec), TNF (100 ng/ml, SEAP induction), Loxoribine (1 mM), or R848 (10 µg/ml). HEK-Blue *Null2-k* cells served as negative control. Results are expressed as mean±SEM. Data were analyzed by one-way ANOVA followed by Dunnett's post hoc test. *$p < 0.05$, **$p < 0.01$, ***$p < 0.001$, ****$p < 0.0001$ *vs*. control ($n = 4$).
